# Novel *KCNQ1* Q234K variant, identified in patients with long QT syndrome and epileptiform activity, induces both gain- and loss-of-function of slowly activating delayed rectifier potassium currents

**DOI:** 10.3389/fphys.2024.1401822

**Published:** 2024-07-19

**Authors:** Tadashi Nakajima, Shuntaro Tamura, Reika Kawabata-Iwakawa, Hideki Itoh, Hiroshi Hasegawa, Takashi Kobari, Shun Harasawa, Akiko Sekine, Masahiko Nishiyama, Masahiko Kurabayashi, Keiji Imoto, Yoshiaki Kaneko, Yosuke Nakatani, Minoru Horie, Hideki Ishii

**Affiliations:** ^1^ Department of Cardiovascular Medicine, Gunma University Graduate School of Medicine, Maebashi, Japan; ^2^ Division of Integrated Oncology Research, Gunma University Initiative for Advanced Research, Maebashi, Japan; ^3^ Division of Patient Safety, Hiroshima University Hospital, Hiroshima, Japan; ^4^ Division of Neurology, Japanese Red Cross Maebashi Hospital, Maebashi, Japan; ^5^ Gunma University, Maebashi, Japan; ^6^ National Institutes of Natural Sciences, Tokyo, Japan; ^7^ Department of Cardiovascular Medicine, Shiga University of Medical Science, Ohtsu, Japan

**Keywords:** epilepsy, epileptiform activity, I_Ks_, *KCNQ1*, long QT syndrome, segment 4, voltage-sensor

## Abstract

**Introduction:**

KCNQ1 and KCNE1 form slowly activating delayed rectifier potassium currents (I_Ks_). Loss-of-function of I_Ks_ by *KCNQ1* variants causes type-1 long QT syndrome (LQTS). Also, some *KCNQ1* variants are reported to cause epilepsy. Segment 4 (S4) of voltage-gated potassium channels has several positively-charged amino acids that are periodically aligned, and acts as a voltage-sensor. Intriguingly, KCNQ1 has a neutral-charge glutamine at the third position (Q3) in the S4 (Q234 position in KCNQ1), which suggests that the Q3 (Q234) may play an important role in the gating properties of I_Ks_. We identified a novel *KCNQ1* Q234K (substituted for a positively-charged lysine) variant in patients (a girl and her mother) with LQTS and epileptiform activity on electroencephalogram. The mother had been diagnosed with epilepsy. Therefore, we sought to elucidate the effects of the *KCNQ1* Q234K on gating properties of I_Ks_.

**Methods:**

Wild-type (WT)-KCNQ1 and/or Q234K-KCNQ1 were transiently expressed in tsA201-cells with KCNE1 (E1) (WT + E1-channels, Q234K + E1-channels, and WT + Q234K + E1-channels), and membrane currents were recorded using whole-cell patch-clamp techniques.

**Results:**

At 8-s depolarization, current density (CD) of the Q234K + E1-channels or WT + Q234K + E1-channels was significantly larger than the WT + E1-channels (WT + E1: 701 ± 59 pA/pF; Q234K + E1: 912 ± 50 pA/pF, *p* < 0.01; WT + Q234K + E1: 867 ± 48 pA/pF, *p* < 0.05). Voltage dependence of activation (VDA) of the Q234K + E1-channels or WT + Q234K + E1-channels was slightly but significantly shifted to depolarizing potentials in comparison to the WT + E1-channels ([V_1/2_] WT + E1: 25.6 ± 2.6 mV; Q234K + E1: 31.8 ± 1.7 mV, *p* < 0.05; WT + Q234K + E1: 32.3 ± 1.9 mV, *p* < 0.05). Activation rate of the Q234K + E1-channels or WT + Q234K + E1-channels was significantly delayed in comparison to the WT + E1-channels ([half activation time] WT + E1: 664 ± 37 ms; Q234K + E1: 1,417 ± 60 ms, *p* < 0.01; WT + Q234K + E1: 1,177 ± 71 ms, *p* < 0.01). At 400-ms depolarization, CD of the Q234K + E1-channels or WT + Q234K + E1-channels was significantly decreased in comparison to the WT + E1-channels (WT + E1: 392 ± 42 pA/pF; Q234K + E1: 143 ± 12 pA/pF, *p* < 0.01; WT + Q234K + E1: 209 ± 24 pA/pF, *p* < 0.01) due to delayed activation rate and depolarizing shift of VDA.

**Conclusion:**

The *KCNQ1* Q234K induced I_Ks_ gain-of-function during long (8-s)-depolarization, while loss of-function during short (400-ms)-depolarization, which indicates that the variant causes LQTS, and raises a possibility that the variant may also cause epilepsy. Our data provide novel insights into the functional consequences of charge addition on the Q3 in the S4 of KCNQ1.

## 1 Introduction

Long QT syndrome (LQTS) is a genetic disorder characterized by QT prolongation on electrocardiogram (ECG) and polymorphic ventricular tachycardia, torsades de pointes, and ventricular fibrillation, leading to syncope and sudden cardiac death (Nakajima et al., 2021). At least 17 causative genes have been identified thus far, although not all appear to cause LQTS (Adler et al., 2020). Most causative genes of LQTS encode cardiac ion channels or their auxiliary subunits. The first three identified genes, which account for approximately 90% of genetically affected LQTS patients, encode α-subunits of cardiac voltage-gated ion channels: *KCNQ1* for type-1 LQTS, *KCNH2* for type-2 LQTS, and *SCN5A* for type-3 LQTS.

*KCNQ1* belongs to the voltage-gated potassium channel superfamily and encodes the pore-forming α-subunit of slowly activating delayed rectifier potassium channels (I_Ks_); KCNQ1 interacts with KCNE1, an auxiliary subunit, and forms I_Ks_ (Barhanin et al., 1996; Sanguinetti et al., 1996). KCNQ1 is expressed in both the heart and brain (Goldman et al., 2009; Rannals et al., 2016). In the heart, loss-of-function of I_Ks_ can prolong cardiac action potential duration (APD), thus prolonging the QT interval on ECG. Multiple loss-of-function mechanisms in I_Ks_, including trafficking defects, producing non-functional channels, altered channel gating properties and a combination thereof, underlie LQTS (Yamashita et al., 2001; Peroz et al., 2008). Notably, the altered gating properties of I_Ks_ can cause both loss- and gain-of-function, thus they can be associated with not only LQTS but also other arrhythmic disorders, such as short QT syndrome and atrial fibrillation (Chen et al., 2003; Bellocq et al., 2004; Hong et al., 2005; Bartos et al., 2011; Hasegawa et al., 2014; Zhou et al., 2019). On the other hand, there have been several reports that *KCNQ1* variants may also be associated with epilepsy as a brain/cerebral phenotype (Goldman et al., 2009; Tiron et al., 2015).

The α-subunits of voltage-gated ion channels have six transmembrane domains: segment (S) 1-S6. The first four transmembrane segments (S1-S4) form a voltage-sensing domain (VSD), the remaining two segments (S5-S6) form a pore domain (PD), and the central PD is surrounded by four VSDs that respond to membrane depolarization. S4 in the VSD typically consists of several positively-charged amino acids (AAs) that are periodically aligned in every three AAs, and is postulated to function as a voltage-sensor (Nakajima et al., 2019; Wu and Larsson, 2020). Therefore, variants in the S4 of genes encoding cardiac voltage-gated ion channels, such as *KCNQ1*, *KCNH2* and *SCN5A*, can confer unique gating properties in each channel, and it is therefore possible that they may confer unique phenotypic manifestations (Nakajima et al., 1999; Hasegawa et al., 2014; Nakajima et al., 2015).

**FIGURE 1:**
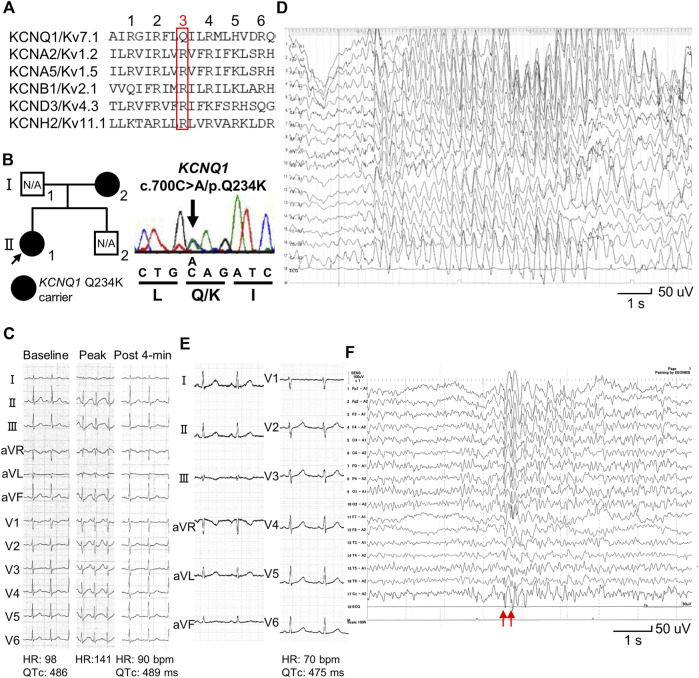
Identification of a *KCNQ1* Q234K variant in patients with long QT syndrome and epileptiform activity on electroencephalogram. **(A)** Alignment of segment 4 (S4) of voltage-gated potassium channels. The third position of amino acids that are periodically aligned is surrounded by a red box. The third position (Q3) of S4 in KCNQ1 corresponds to the position Q234 in KCNQ1. **(B)** Pedigree harboring the *KCNQ1* Q234K variant (left panel). Arrow indicates the proband. Black filled symbols indicate heterozygous carriers of the *KCNQ1* Q234K variant. N/A indicates not assessed. Electropherogram of part of *KCNQ1* exon 5 of the proband (II-1) (right panel). **(C)** 12-lead ECGs of an exercise stress test using a treadmill of the proband (II-1). Those before exercise (baseline) (left panel), at peak exercise (middle panel), and at recovery phase of 4-min (right panel) are shown. **(D)** Electroencephalogram (EEG) of the proband (Ⅱ-1). It showed frequent 3 Hz spike-and-wave complexes lasting several seconds on high amplitude background slow waves. **(E)** 12-lead ECG of the proband’s mother (Ⅰ-2). **(F)** EEG of the proband’s mother (Ⅰ-2). It showed generalized 5–6 Hz spike-and-wave complexes (red arrows), followed by slow waves.

The AA alignment of the S4 of KCNQ1 is unique from that of shaker-like potassium channels (Wu et al., 2010; Sun and MacKinnon, 2017) (Figure 1A). It consists of R1 (arginine at the first position of positively-charged AAs in the S4) (or R228: arginine at position 228 in KCNQ1), R2 (or R231), Q3 (or Q234), R4 (or R237), H5 (or H240), and R6 (or R243). Notably, unlike shaker-like potassium channels, the third position in the S4 of KCNQ1 is not a positively-charged AA but a neutral-charge AA, glutamine (Q3 or Q234) (Figure 1A). This suggests that the neutral-charge at the third position in the S4 of KCNQ1 may play an important role in the gating properties of I_Ks_.

We recently identified a novel *KCNQ1* variant, Q234K (substituted for a positively-charged lysine) (or Q3K), in patients (a girl and her mother) with LQTS and epileptiform activity on electroencephalogram (EEG). The mother had been diagnosed with epilepsy. This attracted our interest in how the variant affects I_Ks_ and clinical phenotypes. Therefore, we sought to elucidate the effects of the *KCNQ1* Q234K variant on the gating properties of KCNQ1 channels and I_Ks_ using patch-clamp techniques.

## 2 Materials and methods

### 2.1 Genetic analysis

This study was approved by the Gunma University Ethical Review Board for Medical Research Involving Human Subjects (approval number: HS 2017–15). Written informed consents of the subjects (the proband and her mother) were obtained from the proband’s mother. The Genomic DNA was extracted from peripheral blood lymphocytes. Target panel sequencing of 72 genes, including inherited arrhythmia syndrome-related genes, was performed using the proband’s sample as previously described (Nakajima et al., 2020a). Confirmation of nucleotide substitutions in the proband and her mother was performed using Sanger sequencing. Exon 5 of *KCNQ1* (NM_000218.3) was analyzed as previously described (Imai et al., 2014).

### 2.2 Mutagenesis and heterologous expression

Full-length cDNA encoding human wild-type (WT)-KCNQ1 subcloned into a pIRES2-EGFP expression vector (WT-KCNQ1 cDNA) was kindly provided by Prof. Jacques Barhanin (Laboratories of Excellence, Ion Channel Science and Therapeutics, Nice, France). Q234K-KCNQ1 cDNA was constructed using a Quick Change II XL site-directed mutagenesis kit (Agilent Technologies, Santa Clara, CA, United States). Full-length cDNA encoding human KCNE1 subcloned into the pCDNA3.1 expression vector (KCNE1 cDNA) was obtained as described previously (Wu et al., 2014).

For electrophysiological characterization of WT-KCNQ1 (WT alone) channels and Q234K-KCNQ1 (Q234K alone) channels, 0.5 μg of WT-KCNQ1 cDNA alone or 0.5 μg of Q234K-KCNQ1 cDNA alone was transiently transfected into tsA201-cells using Lipofectamine 2000 (Invitrogen, Carlsbad, CA, United States). For electrophysiological characterization of WT-KCNQ1+KCNE1 (WT + E1) channels and Q234K-KCNQ1+KCNE1 (Q234K + E1) channels, 0.5 μg of WT-KCNQ1 cDNA or 0.5 μg of Q234K-KCNQ1 cDNA in combination with 0.5 μg KCNE1 cDNA was transiently transfected. For electrophysiological characterization of WT-KCNQ1+Q234K-KCNQ1+KCNE1 (WT + Q234K + E1) channels, 0.25 μg of WT-KCNQ1 cDNA plus 0.25 μg of Q234K-KCNQ1 cDNA in combination with 0.5 μg KCNE1 cDNA was transiently transfected. Transfected cells were maintained in DMEM at 37°C for 42–54 h before current recordings as described previously (Nakajima et al., 2022). Cells that exhibited green fluorescence were chosen for the current recordings.

### 2.3 Electrophysiology

Membrane currents were recorded using whole-cell patch-clamp techniques at room temperature (23°C–25°C). The bath solution contained (in mmol/L) 140 NaCl, 4 KCl, 2 CaCl_2_, 1 MgCl_2_, 5 glucose and 10 HEPES (pH 7.4 with NaOH), and the pipette solution contained (in mmol/L) 140 KAsp, 1 MgCl_2_, 4 MgATP, 10 EGTA and 10 HEPES (pH 7.3 with KOH). The electrode resistance ranged from 2.0 to 3.0 MΩ. Data acquisition was carried out using an Axopatch 200B amplifier and pCLAMP10.3 software (Molecular Devices, Sunnyvale, CA, United States). Currents were acquired at 20–50 kHz, and low pass-filtered at 5 kHz using an analog-to-digital interface (Digidata 1440A acquisition system, Molecular Devices). Current densities at each test potential were obtained by dividing the expressed currents by cell capacitance.

All pulse protocols are shown in each of Figures or Figure legends. The voltage dependence of activation was fitted with a Boltzmann function of the following form: y = 1–1/{1+exp[(V_m_-V_1/2_)/*K*]}, where y is the relative current, V_m_ is the membrane potential, V_1/2_ is the voltage at which half of the channels are available to open, and *K* is the slope factor. The time course of activation was fitted with a single exponential function of the following form: *I*(t)/I_max_ = A_0_+A_1_exp(-t/τ), and the time course of deactivation was fitted with a single exponential function of the following form: *I*(t)/I_max_ = A_0_+A_1_[1-exp(-t/τ)], where A and τ refer to the amplitude and time constant, respectively, I refers to the current, and t refers to the time.

To avoid potential endogenous current contamination, recordings from the cells exhibiting peak outward current amplitudes of <0.8 nA were excluded from the analyses.

### 2.4 Simulation study

In order to understand how the changes of the channel kinetics or current density in I_Ks_ might affect APD or QT interval, we mimicked the experimental data for the WT or the Q234K model and simulated action potentials across the transmural myocardium or ECG (Itoh et al., 2009; Kojima et al., 2020). O’Hara and Rudy model was available for this simulation (O'Hara et al., 2011), and we searched for appropriate parameters according to the experimental data under the Q234K heterozygous condition. For the analysis of I_Ks_, we used the experimental data in which KCNQ1 would be co-expressed with KCNE1. We modified a parameter associated with Hodgkin-Huxley model in I_Ks_ and simulated the Q234K + WT + E1 model as shown below.

$$\left(WT+E1\right);\;\tau x,S2=\frac{1}{0.01\times \exp\left(\frac{V-50}{20}\right)+0.0193\times \exp\;\left(-\frac{\left(V+66.54\right)}{31}\right)}$$

$$\left(Q234K+WT+E1\right);\;\tau x,S2=\frac{5}{0.01\times \exp\left(\frac{V-50}{20}\right)+0.0193\times \exp\;\left(-\frac{\left(V+66.54\right)}{31}\right)}$$

To simulate the transmural myocardium and pseudo-ECG, we linearly connected 165 myocardial cells through epicardial (1–60), mid-myocardial (61–105), and endocardial cells (106–165). The cells were stimulated in the endocardial cell for 3 min at the cycle length of 1,000 ms. The pseudo-ECG was simulated using Gima and Rudy model (Gima and Rudy, 2002).

### 2.5 Statistical analysis

All data are expressed as mean ± standard error, and statistical comparisons were tested using the unpaired Student’s t-test with *p* < 0.05 considered to be statistically significant. In some figures, the standard error bars are smaller than the data symbols.

## 3 Results

### 3.1 Identification of a novel *KCNQ1* Q234K variant in patients with LQTS and epileptiform activity on EEG

A 13-year-old female (II-1-1) (Figure 1B) was referred to our institution because QT prolongation on her ECG had been pointed out at medical check-up in junior high school. Her 12-lead ECG showed QTc prolongation (QTc: 486 ms) (Figure 1C). She experienced an episode of syncope several months ago, but the cause was unknown. Transthoracic echocardiography revealed no structural heart diseases. An exercise stress test using a treadmill did not induce any ventricular arrhythmias, but induced QTc prolongation at peak exercise and at recovery phase of 4-min (QTc: 489 ms) (Figure 1C). Her EEG showed frequent spike-and-wave complexes (Figure 1D), although there was no epileptic seizure while recording EEG.

12-lead ECG of her mother (I-2) (Figure 1B) also showed QTc prolongation (QTc: 475 ms) (Figure 1E). She had experienced several episodes of syncope since she was 1 year of age. Prodromal symptoms, such as blinking her eyes, were present before syncope, and her EEGs recorded repeatedly showed spike-and-wave complexes, which led to a diagnosis of epilepsy at 3 years of age. Sodium valproate had been prescribed for many years, but she discontinued treatment on her own judgment several years ago. She had been asymptomatic since she was 4 years of age. Her EEG recorded at age 39 also showed spike-and-wave complexes (Figure 1F).

Target panel sequencing of 72 genes, including inherited arrhythmia syndrome-related genes, was performed in the proband (II-1) (Figure 1B). A novel *KCNQ1* Q234K variant was identified and validated using Sanger sequencing (Figure 1B). Sanger sequencing of the proband’s mother (I-2) (Figure 1B) revealed that she harbored the same variant. This variant was not found in gnomAD (https://gnomad.broadinstitute.org/) and ClinVar (https://www.ncbi.nlm.nih.gov/clinvar/), and was deleterious in SIFT (https://sift.bii.a-star.edu.sg/) and probably damaging in PolyPhen-2 (http://genetics.bwh.harvard.edu/pph2/).

### 3.2 Gating abnormalities of the Q234K-KCNQ1 (Q234K alone) channels

**FIGURE 2:**
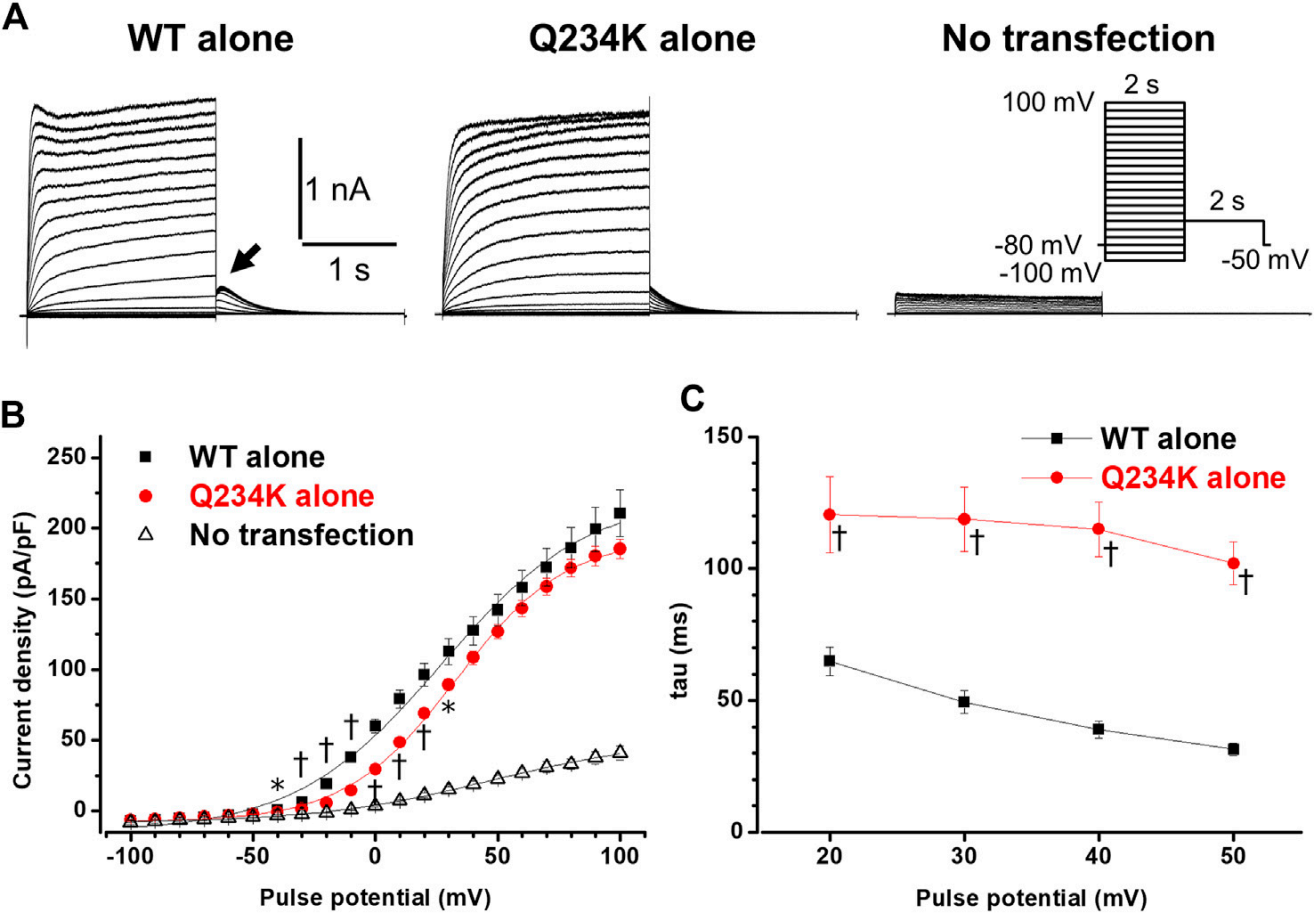
Gating abnormalities of the Q234K alone channels obtained by a 2-s depolarization pulse protocol. **(A)** Representative currents, obtained by the pulse protocol in the inset, from a cell transfected with wild-type (WT) KCNQ1 cDNA alone (WT alone) (left panel), a cell transfected with Q234K KCNQ1 cDNA alone (Q234K alone) (middle panel), and a non-transfected cell (No transfection) (right panel). The arrow in the left panel indicates the hook. **(B)** The current-voltage relationships, measured at the end of 2-s depolarizing pulses, of the WT alone (black filled squares, n = 9), Q234K alone (red filled circles, n = 10) and no transfection (black open triangles, n = 6). * indicates *p* < 0.05 vs WT + E1 and † indicates *p* < 0.01 vs WT + E1. **(C)** The time constants (taus) of initial 200-ms activating currents of the WT alone (black filled squares, n = 9) and Q234K alone (red filled circles, n = 10) fitted with a single exponential function between 20 mV and 50 mV in 10 mV increments.

We first compared the electrophysiological properties of the WT alone channels and Q234K alone channels. The current density of the Q234K alone channels, measured at the end of a 2-s depolarizing potential of 100 mV, was not different from that of the WT alone channels (Table 1) (Figures 2A,B), indicating that trafficking of the Q234K alone channels might not be impaired. However, the voltage dependence of activation (VDA) of the Q234K alone channels was significantly shifted to positive potentials (≈5.8 mV) in comparison to the WT alone channels (Table 1) (Figure 2B).

**TABLE 1:** Activation parameters of the WT alone and Q234K alone obtained by a 2-s depolarizing pulse protocol.

	CD (pA/pF)at 100 mV	V_1/2_(mV)	*K* (mV)	Activation tau (ms)			
				at 20 mV	at 30 mV	at 40 mV	at 50 mV
WT alone (n = 9)	211 ± 16	27.4 ± 2.0	30.2 ± 0.8	65 ± 5	49 ± 4	39 ± 3	31 ± 2
Q234K alone (n = 10)	185 ± 7	33.2 ± 1.2*	22.7 ± 0.5†	120 ± 14†	119 ± 12†	115 ± 10†	102 ± 8†

WT, wild-type; CD, current density; V_1/2_, voltage at which half of the channels are available to open; *K*, slope factor; tau, time constant; *p < 0.05 vs WT alone; †*p* < 0.01 vs WT alone.

The activating currents were fitted with a single exponential function. The time constants of the Q234K alone channels were significantly larger than those of the WT alone channels, indicating that the Q234K alone channels exhibited a delay of the activation rate in comparison to the WT alone channels (Table 1) (Figures 2A,C). Notably, the WT alone channels exhibited inactivation at higher depolarizing potentials while the Q234K alone channels did not exhibit apparent inactivation (Figure 2A). The absence of inactivation in the Q234K alone channels might, at least in part, contribute to the delayed activation rate in comparison to the WT alone channels.

**FIGURE 3:**
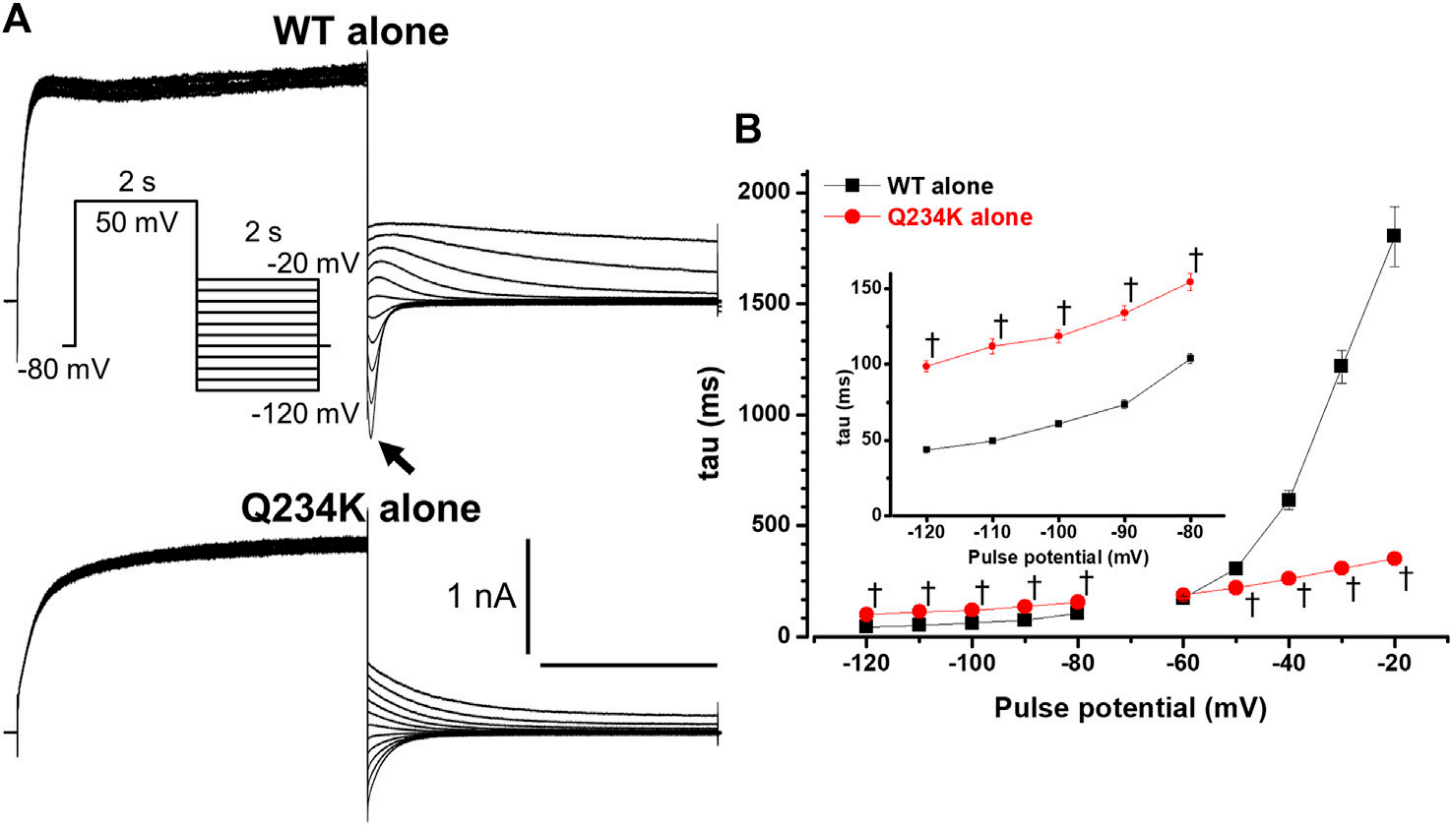
The *KCNQ1* Q234K mutation affected deactivation rate in a voltage-dependent manner. **(A)** Representative currents of the WT alone (upper panel) and Q234K alone (lower panel) obtained by the pulse protocol in the inset. The arrow in the upper panel indicates the hook. **(B)** The time constants (taus) of deactivating currents of the WT alone (black filled squares, n = 11) and Q234K alone (red filled circles, n = 12) fitted with a single exponential function between −120 mV and −20 mV in 10 mV increments. The tau at −70 mV was deleted because −70 mV was close to the reversal potential. The inset shows an expanded scale of taus between −120 mV and −80 mV † indicates *p* < 0.01 vs WT + E1.

Tail currents of the WT alone channels, recorded at −50 mV after depolarizing pulses, exhibited hooks (Figure 2A), the initial component of which indicated that the current increase may have resulted from recovery from inactivation, as described previously (Tristani-Firouzi and Sanguinetti, 1998; Franqueza et al., 1999), whereas those of the Q234K alone channels did not exhibit apparent hooks (Figure 2A). The hooks were also present at repolarizing potentials after depolarization in the WT alone channels, but not in the Q234K alone channels (Figure 3A). The presence of the hook in the tail currents of the WT alone channels suggested that these channels inactivated during depolarization and recovered from inactivation during repolarization, which is compatible with the notion that the WT alone channels inactivate during depolarization (Tristani-Firouzi and Sanguinetti, 1998). On the other hand, the lack of a hook in the tail currents of the Q234K alone channels suggested that these channels did not inactivate during depolarization or that they recovered from inactivation very quickly. The disappearance of the hook has also been observed in other variants of KCNQ1 (Franqueza et al., 1999; Moreno et al., 2017).

The deactivation rate was assessed by fitting deactivating currents with a single exponential function. In the WT alone channels, an initial increase in the hook was not included in the fitting procedure (Figure 3A). Intriguingly, the time constants of the Q234K alone channels were significantly larger at hyperpolarizing potentials (below the reversal potential) but were significantly smaller at depolarizing potentials (over the reversal potential) in comparison to the WT alone channels (Table 2) (Figure 3B), indicating that the deactivation rate of the Q234K alone channels was decelerated at hyperpolarizing potentials but accelerated at depolarizing potentials.

**TABLE 2:** Deactivation time constants of the WT alone and Q234K alone.

	Deactivation tau (ms)									
	−120 mV	−110 mV	−100 mV	−90 mV	−80 mV	−60 mV	−50 mV	−40 mV	−30 mV	−20 mV
WT alone (n = 11)	44 ± 2	49 ± 2	61 ± 2	74 ± 3	104 ± 3	172 ± 9	305 ± 18	615 ± 43	1,217 ± 74	1,805 ± 134
Q234K alone (n = 12)	99 ± 4†	112 ± 5†	119 ± 4†	134 ± 5†	154 ± 6†	187 ± 7	220 ± 7†	261 ± 8†	307 ± 9†	351 ± 10†

WT, wild-type; tau, time constant; †*p* < 0.01 vs WT alone.

### 3.3 Gating abnormalities of the Q234K-KCNQ1+KCNE1 (Q234K + E1) channels

**FIGURE 4:**
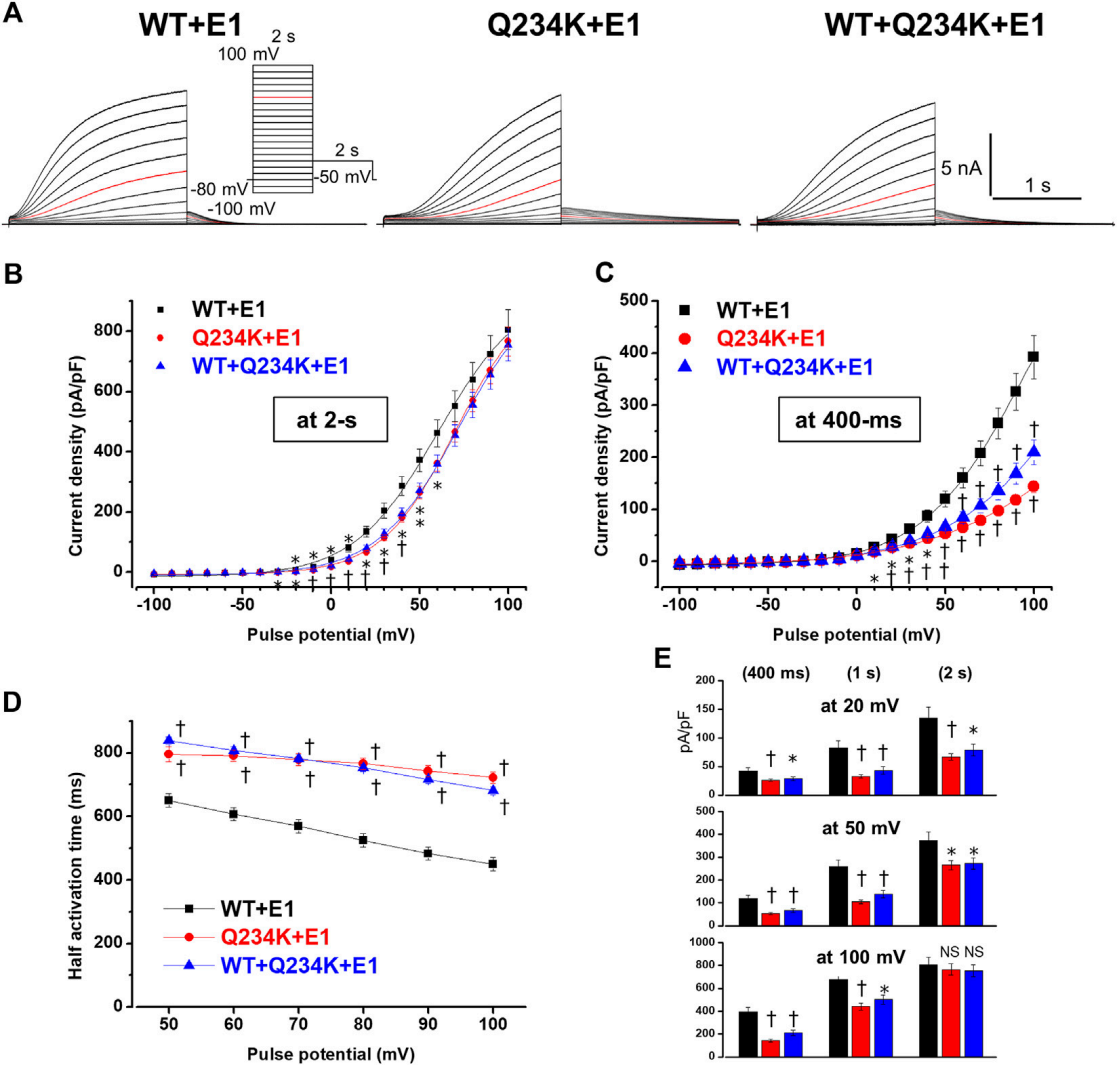
Gating abnormalities of the Q234K + E1 channels and WT + Q234K + E 1 channels revealed by the 2-s depolarization pulse protocol. **(A)** Representative currents of WT + E1 channels (left panel), Q234K + E1 channels (middle panel) and WT + Q234K + E1 channels (right panel) obtained by the pulse protocol in the inset. Red current tracings indicate the currents recorded at a depolarizing potential of 50 mV. **(B)** and **(C)** The current-voltage relationships of the WT + E1 channels (black filled squares, n = 14), Q234K + E1 channels (red filled circles, n = 14), and WT + Q234K + E1 channels (blue filled triangles, n = 15) at the end of 2-s **(B)** and 400-ms **(C)** depolarizing potentials. * indicates *p* < 0.05 vs WT + E1 and † indicates *p* < 0.01 vs WT + E1. **(D)** The half activation time during 2-s depolarizing pulses of the WT + E1 channels (black filled squares, n = 14), Q234K + E1 channels (red filled circles, n = 14), and WT + Q234K + E1 channels (blue filled triangles, n = 15). **(E)** The current densities of the WT + E1 channels (black bars, n = 14), Q234K + E1 channels (red bars, n = 14), and WT + Q234K + E1 channels (blue bars, n = 15) measured at various depolarizing potentials (20 mV, 50 mV, and 100 mV) and various pulse durations (400-ms, 1-s, and 2-s).

The interaction of KCNE1 with KCNQ1 forms I_Ks_ and dramatically affects WT alone channels. Therefore, we compared the electrophysiological properties of the WT-KCNQ1+KCNE1 (WT + E1) channels and Q234K-KCNQ1+KCNE1 (Q234K + E1) channels. The current density, measured at the end of the 2-s depolarizing potential of 100 mV, of the Q234K + E1 channels was not different from that of the WT + E1 channels (Table 3) (Figures 4A,B). The VDA of the Q234K + E1 channels was significantly shifted to positive potentials (≈8.5 mV) in comparison to the WT + E1 channels (Table 3) (Figure 4B).

**TABLE 3:** Activation parameters and current densities, measured at various depolarizing potentials and durations, of the WT + E1, Q234K + E1, and WT + Q234K + E1 obtained by a 2-s depolarizing pulse protocol.

		V_1/2_ (mV)	*K* (mV)	HAT (ms) at 100 mV
WT + E1 (n = 14)		62.8 ± 2.2	25.4 ± 1.2	449 ± 21
Q234K + E1 (n = 14)		71.3 ± 1.1†	21.8 ± 0.6†	722 ± 18†
WT + Q234K + E1 (n = 15)		72.5 ± 0.7†	23.6 ± 0.6	681 ± 13†

		Current density (pA/pF)		
		at 400-ms	at 1-s	at 2-s
WT + E1	at 20 mV	42 ± 6	83 ± 12	135 ± 19
Q234K + E1		26 ± 2†	33 ± 3†	67 ± 6†
WT + Q234K + E1		28 ± 4*	43 ± 6†	79 ± 11*
WT + E1	at 50 mV	119 ± 15	259 ± 29	372 ± 38
Q234K + E1		53 ± 4†	105 ± 9†	265 ± 21*
WT + Q234K + E1		66 ± 9†	138 ± 17†	272 ± 25*
WT + E1	at 100 mV	392 ± 42	677 ± 60	805 ± 66
Q234K + E1		143 ± 12†	440 ± 32†	767 ± 48
WT + Q234K + E1		209 ± 24†	502 ± 41*	754 ± 53

WT, wild-type; E1, KCNE1; V_1/2_, voltage at which half of the channels are available to open; *K*, slope factor; HAT, half activation time; * *p* < 0.05 vs WT + E1; †*p* < 0.01 vs WT + E1.

Because activating currents during depolarizing pulses for both channels could not be fitted with a single or double exponential function, we assessed the half activation time (HAT) during the 2-s depolarizing pulses. The HAT of the Q234K + E1 channels was significantly larger than that of the WT + E1 channels (Table 3) (Figures 4A,D), indicating that the activation rate of the Q234K + E1 channels was significantly decelerated in comparison to the WT + E1 channels.

We also assessed the current densities at 2-s, 1-s, and 400-ms depolarizing pulses of various depolarizing potentials (20 mV, 50 mV, and 100 mV). Although the current density of the Q234K + E1 channels, measured at the 2-s depolarizing potential of 100 mV, was comparable to that of the WT + E1 channels as mentioned above, those of the Q234K + E1 channels, measured at the 2-s depolarizing potentials of 20 mV and 50 mV, were smaller than those of the WT + E1 channels, mainly due to the depolarizing shift of the VDA. Notably, the current densities, measured at 400-ms and 1-s depolarizing potentials of 100 mV, of the Q234K + E1 channels were significantly smaller than those of the WT + E1 channels (Table 3) (Figures 4C,E), mainly due to the delayed activation rate in the Q234K + E1 channels. On the other hand, the current densities of the Q234K + E1 channels, measured at 400-ms and 1-s depolarizing potentials of 50 mV and 20 mV, were smaller than those of the WT + E1 channels (Table 3) (Figures 4C,E), due to both the depolarizing shift of the VDA and delayed activation rate in the Q234K + E1 channels.

**FIGURE 5:**
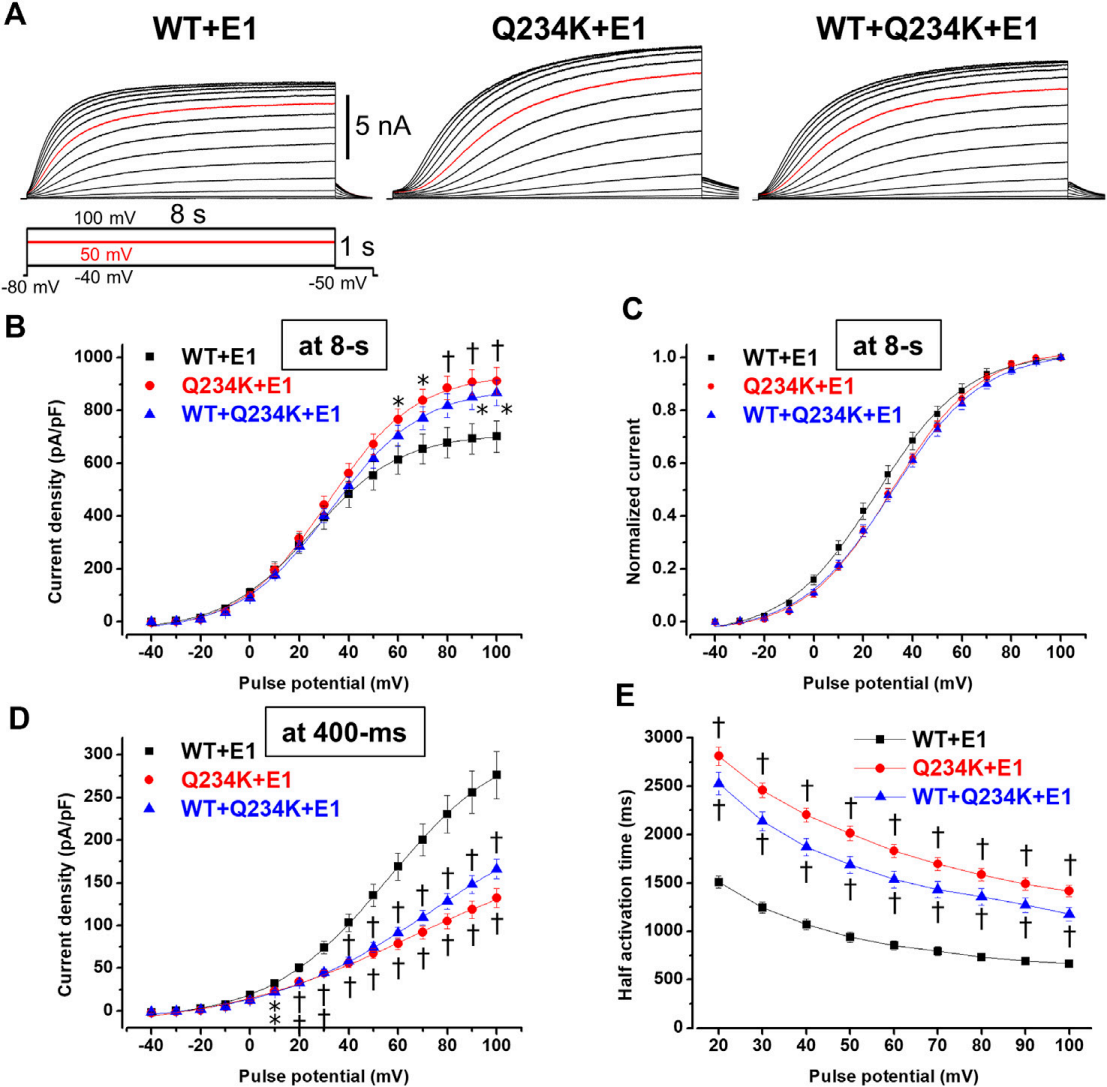
Gating abnormalities of the Q234K + E1 channels and WT + Q234K + E1 channels revealed by an 8-s depolarization pulse protocol. **(A)** Representative currents of the WT + E1 channels (left panel), Q234K + E1 channels (middle panel), and WT + Q234K + E1 channels (right panel) obtained by the pulse protocol in the inset. 8-s depolarizing pulses were applied from −40 mV to 100 mV in 10 mV increments. Red current tracings indicate currents obtained at a depolarizing potential of 50 mV. **(B)** The current-voltage relationships of the WT + E1 channels (black filled squares, n = 16), Q234K + E1 channels (red filled circles, n = 17), and WT + Q234K + E1 channels (blue filled triangles, n = 17) at the end of 8-s depolarizing potentials. * indicates *p* < 0.05 vs WT + E1 and † indicates *p* < 0.01 vs WT + E1. **(C)** Normalized current-voltage relationships of **(B)**. **(D)** The current-voltage relationships of the WT + E1 channels (black filled squares, n = 16), Q234K + E1 channels (red filled circles, n = 17), and WT + Q234K + E1 channels (blue filled triangles, n = 17) at the end of 400-ms depolarizing potentials. **(E)** Half activation times of the WT + E1 channels (black filled squares, n = 16), Q234K + E1 channels (red filled circles, n = 17), and WT + Q234K + E1 channels (blue filled triangles, n = 17).

Because 2-s depolarizing pulses appeared to be insufficient to fully activate both channels, 8-s depolarizing pulses were employed. The current density, measured at the end of the 8-s depolarizing potential of 100 mV, of the Q234K + E1 channels was significantly larger than that of the WT + E1 channels (Table 4) (Figures 5A,B). The VDA of the Q234K + E1 channels was still slightly but significantly shifted to positive potentials (≈6.2 mV) in comparison to the WT + E1 channels (Table 4) (Figure 5C).

**TABLE 4:** Activation parameters of the WT + E1, Q234K + E1, and WT + Q234K + E1 obtained by an 8-s depolarizing pulse protocol.

	at 8-s				at 400-ms
	CD (pA/pF) at 100 mV	V_1/2_ (mV)	*K* (mV)	HAT (ms) at 100 mV	CD (pA/pF) at 100 mV
WT + E1 (n = 16)	701 ± 59	25.6 ± 2.6	17.5 ± 1.1	664 ± 37	276 ± 28
Q234K + E1 (n = 17)	912 ± 50†	31.8 ± 1.7*	17.5 ± 0.5	1,417 ± 60†	132 ± 11†
WT + Q234K + E1 (n = 17)	867 ± 48*	32.3 ± 1.9*	18.3 ± 0.6	1,177 ± 71†	166 ± 11†

WT, wild-type; E1, KCNE1; CD, current density; V_1/2_, voltage at which half of the channels are available to open; *K*, slope factor; HAT, half activation time; * *p* < 0.05 vs WT + E1; †*p* < 0.01 vs WT + E1.

In contrast, the current density, measured at a 400-ms depolarizing potential of 100 mV, of the Q234K + E1 channels was significantly smaller than that of the WT + E1 channels (Table 4) (Figures 5A,D), as obtained by the 2-s depolarizing pulse protocol. The HAT of the Q234K + E1 channels was significantly larger than that of the WT + E1 channels (Table 4) (Figure 4E), indicating that the activation rate of the Q234K + E1 channels was significantly slower than that of the WT + E1 channels, as obtained by the 2-s depolarizing pulse protocol. Like these, using the 8-s depolarizing pulse protocol, we obtained almost the same results as the 2-s depolarizing pulse protocol, except for an increased current density in the Q234K + E1 channels.

**FIGURE 6:**
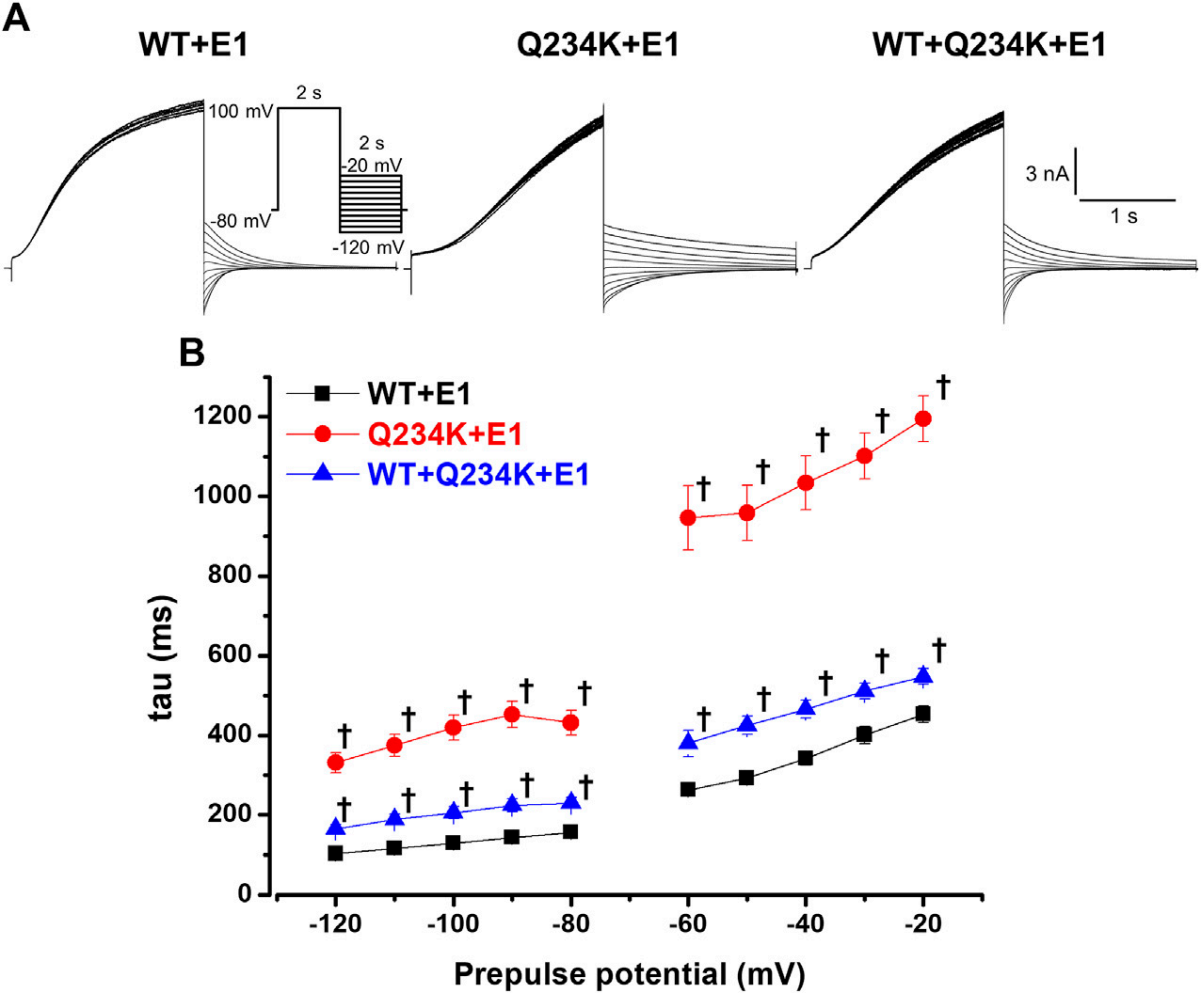
Delayed deactivation rates in the Q234K + E1 channels and WT + Q234K + E1 channels. **(A)** Representative currents of the WT + E1 (left panel), Q234K + E1 (middle panel), and WT + Q234K + E1 (right panel) obtained by the pulse protocol in the inset. **(B)** Time constants (taus) of deactivating currents of the WT + E1 channels (black filled squares, n = 13), Q234K + E1 channels (red filled circles, n = 12), and WT + Q234K + E1 channels (blue filled triangles, n = 14) fitted with a single exponential function between −120 mV and −20 mV in 10 mV increments. The tau at −70 mV was deleted because −70 mV was close to the reversal potential. † indicates *p* < 0.01 vs WT + E1.

The deactivation rates were assessed by fitting deactivating currents during repolarizing pulses with a single exponential function (Figure 6A). The time constants of the Q234K + E1 channels were significantly larger than those of the WT + E1 channels (Table 5) (Figure 6B), indicating that the Q234K + E1 channels caused a significant delay of the deactivation rate.

**TABLE 5:** Deactivation time constants of the WT + E1, Q234K + E1, and WT + Q234K + E1.

	Deactivation tau (ms)									
	−120 mV	−110 mV	−100 mV	−90 mV	−80 mV	−60 mV	−50 mV	−40 mV	−30 mV	−20 mV
WT + E1 (n = 13)	103 ± 4	116 ± 5	130 ± 5	144 ± 5	157 ± 5	263 ± 13	293 ± 15	343 ± 18	401 ± 22	453 ± 20
Q234K + E1 (n = 12)	332 ± 25†	375 ± 28†	450 ± 31†	453 ± 34†	432 ± 31†	946 ± 80†	958 ± 70†	1,034 ± 67†	1,101 ± 58†	1,194 ± 58†
WT + Q234K + E1 (n = 14)	165 ± 11†	189 ± 14†	206 ± 15†	224 ± 16†	230 ± 14†	380 ± 33†	425 ± 23†	466 ± 22†	511 ± 20†	548 ± 19†

WT, wild-type; E1, KCNE1; tau, time constant; †*p* < 0.01 vs WT + E1.

### 3.4 Gating abnormalities of the WT-KCNQ1+Q234K-KCNQ1+KCNE1 (WT + Q234K + E1) channels

Since the patients in this study harbored the *KCNQ1* Q234K variant in a heterozygous manner, we characterized the electrophysiological properties of the WT-KCNQ1+Q234K-KCNQ1+KCNE1 (WT + Q234K + E1) channels.

At the end of the 8-s depolarizing potential of 100 mV, the current density of the WT + Q234K + E1 channels was significantly larger than that of the WT + E1 channels (Table 4) (Figures 5A,B). The VDA of the WT + Q234K + E1 channels was slightly but significantly shifted to positive potentials (≈6.7 mV) in comparison to the WT + E1 channels (Table 4) (Figure 5C). The HATs, obtained by both the 2-s and 8-s depolarizing pulse protocols, of the WT + Q234K + E1 channels were significantly larger than those of the WT + E1 channels (Tables 3 and 4) (Figures 4A,D, 5A,E), indicating that the activation rate of the WT + Q234K + E1 channels was significantly slower than the WT + E1 channels. Using both the 2-s and 8-s depolarizing pulse protocols, the current densities, measured at 400-ms depolarizing potential of 100 mV, of the WT + Q234K + E1 channels were significantly smaller than those of the WT + E1 channels (Tables 3 and 4) (Figures 4A,C, 5A,D).

The time constants of deactivation of the WT + Q234K + E1 channels were significantly larger than those of the WT + E1 channels (Table 5) (Figure 6B), indicating that the deactivation rate of the WT + Q234K + E1 channels was slower than the WT + E1 channels.

### 3.5 Impaired frequency-dependent accumulation of the Q234K + E1 channels and the WT + Q234K + E1 channels

**FIGURE 7:**
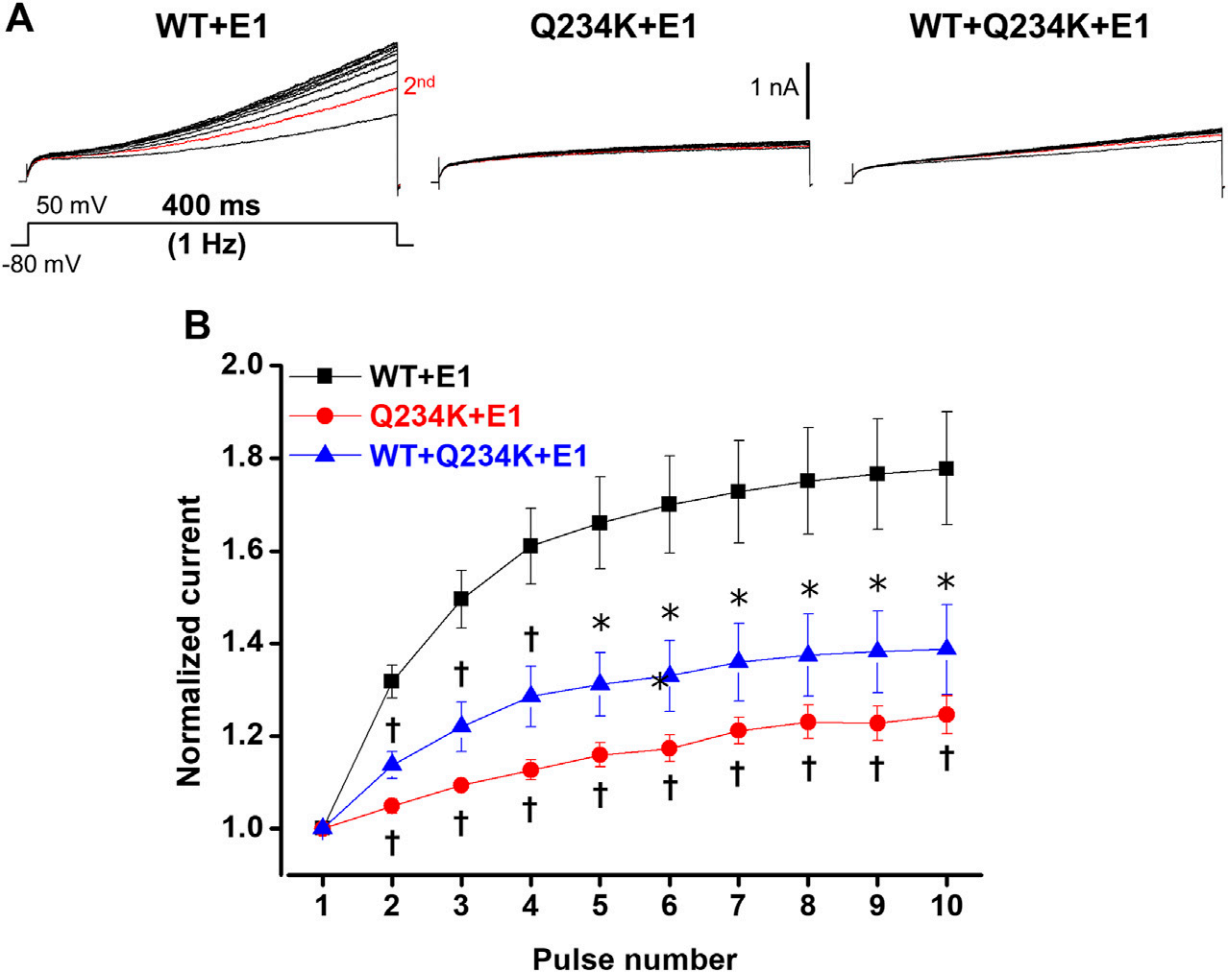
Impaired frequency-dependent accumulation of the Q234K + E1 channels and WT + Q234K + E1 channels. **(A)** Representative currents of the WT + E1 channels (left panel), Q234K + E1 channels (middle panel), and WT + Q234K + E1 channels (right panel) obtained by the pulse protocol in the inset. Red current tracings indicate currents obtained by the second pulse. **(B)** Normalized current amplitudes at 400-ms depolarizing pulse of 50 mV obtained by 10 times repeated pulses at 1 Hz * indicates *p* < 0.05 vs WT + E1 and † indicates *p* < 0.01 vs WT + E1.

The I_Ks_ (WT + E1 channels) have been reported to display frequency-dependent current accumulation (Romey et al., 1997). Therefore, we examined whether the Q234K + E1 channels and WT + Q234K + E1 channels display frequency-dependent current accumulation during the physiological range of ventricular APD. For this purpose, repeated 400-ms depolarizing pulses at 1 Hz were applied. The WT + E1 channels displayed frequency-dependent current accumulation, while the Q234K + E1 channels and the WT + Q234K + E1 channels displayed weak frequency-dependent current accumulation in comparison to the WT + E1 channels (Table 6) (Figures 7A,B).

**TABLE 6:** Normalized peak current amplitudes of the WT + E1, Q234K + E1, and WT + Q234K + E1 obtained by 400-ms repetitive depolarizing pulses of 50 mV applied by 1 Hz.

Pulse number	Normalized peak current amplitudes								
	2	3	4	5	6	7	8	9	10
WT + E1 (n = 8)	1.32 ± 0.04	1.49 ± 0.06	1.61 ± 0.08	1.66 ± 0.10	1.70 ± 0.10	1.73 ± 0.11	1.75 ± 0.11	1.77 ± 0.12	1.78 ± 0.12
Q234K + E1 (n = 9)	1.05 ± 0.02†	1.09 ± 0.01†	1.13 ± 0.02†	1.16 ± 0.03†	1.17 ± 0.03†	1.21 ± 0.03†	1.23 ± 0.04†	1.23 ± 0.04†	1.25 ± 0.04†
WT + Q234K + E1 (n = 7)	1.13 ± 0.03†	1.22 ± 0.05†	1.22 ± 0.05†	1.31 ± 0.07†	1.33 ± 0.08†	1.36 ± 0.09†	1.37 ± 0.09†	1.38 ± 0.09†	1.39 ± 0.09†

WT, wild-type; †*p* < 0.01 vs WT + E1.

### 3.6 Simulation study

**FIGURE 8:**
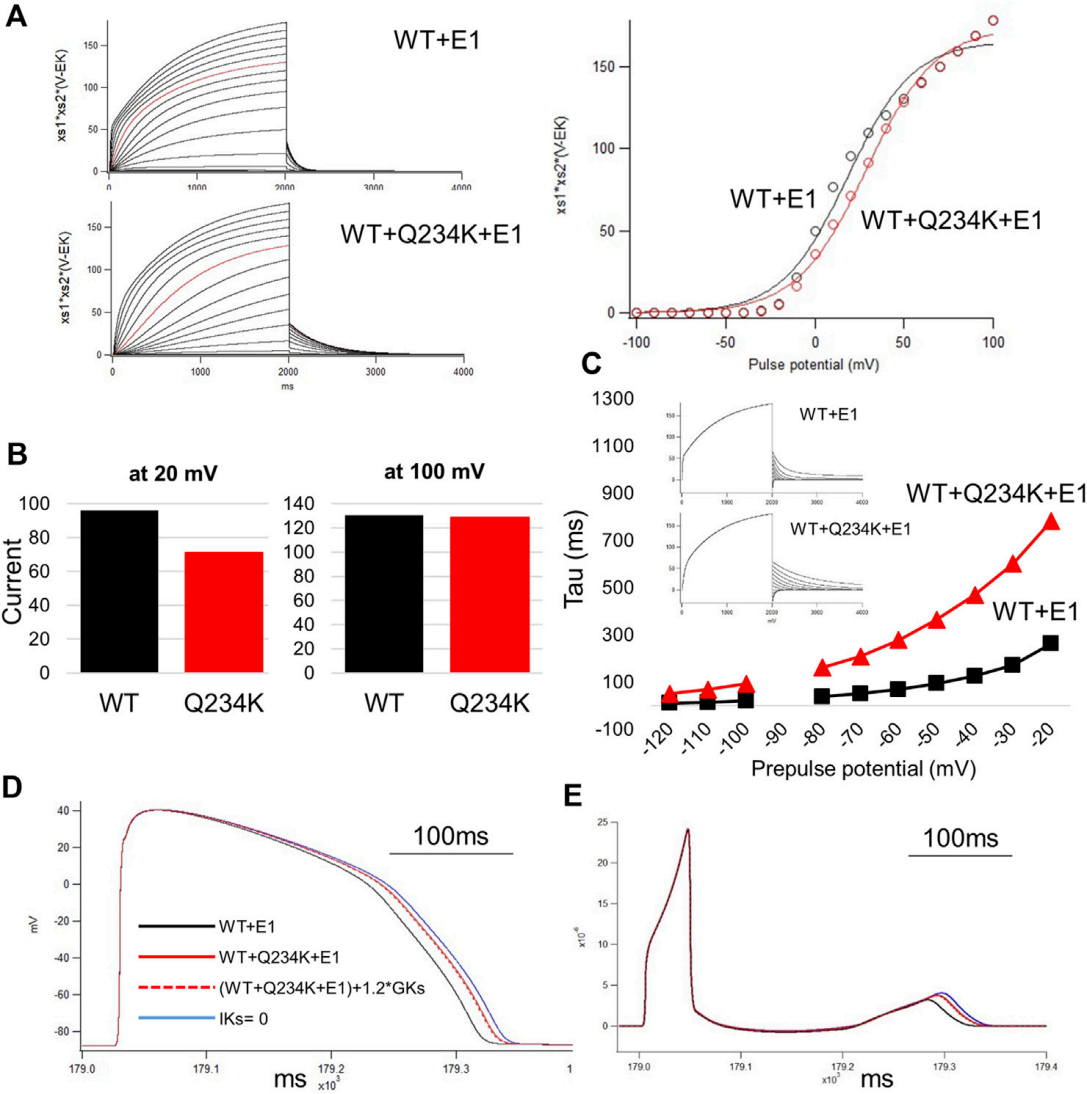
The simulation study of the WT and Q234K heterozygous model. The O’Hara and Rudy model was available in this study. **(A)** Current-voltage relationships for amplitudes of steady-state currents at the end of 2-s depolarizing pulses. Current traces were either WT + E1 or WT + Q234K + E1 and red lines were recorded at a depolarizing pulse of 50 mV (left panel). Black open circles = WT + E1, red open circles = WT + Q234K + E1. The voltage clamp protocol was same as shown in Figure 4A. **(B)** The currents at the depolarizing pulse of 20 mV or 100 mV. WT = WT + E1, Q234K = WT + Q234K + E1. The current of the Q234K model was smaller than the WT at the depolarizing pulse of 20 mV while currents were similar at the depolarizing pulse of 100 mV. **(C)** The time constant of deactivation. The voltage clamp protocol was same as shown in Figure 6A and the insets were current traces at those times. **(D)** The simulated action potentials in mid-myocardial cell which was 90th from the epicardium. Black line = WT + E1, red solid line = WT + Q234K + E1 without the modification of the current density, red dotted line = WT + Q234K + E1 with the increased current density, and blue line = a model with abolished I_Ks_. **(E)** Pseudo-ECGs of WT or Q234K-heterozygous carrier. The QT interval of Q234K heterozygous model were between WT and the model without I_Ks_. The modification of the current density in the Q234K model almost did not affect QT interval.

In order to elucidate how I_Ks_ with the Q234K would affect the action potential or QT interval, we focused on three characteristics of the Q234K channel following the experimental data; the shift of the voltage dependence of activation toward the depolarizing side, the slower deactivation time curse, and the larger current density. The modification of the time constant, τx,S2 , alone was able to mimic the characteristics of activation and deactivation in the Q234K channel (Figures 8A–C). The V_1/2_ for activation was 18.3 mV for WT and 28.7 mV for the Q234K heterozygous model. As shown in Figures 8D,E, the Q234K heterozygous model without the modification of current density had prolonged the APD and QT interval which were intermediate between the WT and the model without I_Ks_ current like type-1 LQTS. The APD or QT interval in the pseudo-ECG failed to be shortened even though the current density would be increased to 1.2 times of WT corresponding to the experimental data, and the simulated action potential and ECG were almost similar to the Q234K heterozygous model without the modification of current density.

## 4 Discussion

### 4.1 Role of the S4 in voltage-gated potassium channels and unique alignments of the S4 of KCNQ1

The S4 of α-subunits of voltage-gated potassium channels typically consists of several positively-charged AAs aligned periodically in every three AAs, and is postulated to function as a voltage-sensor. It is widely accepted that the S4 in the VSD senses depolarizing voltage changes across the membrane and moves outwards within the membrane. This outward movement leads to conformational changes in the VSD that trigger structural rearrangements in the PD (VSD-PD coupling or electromechanical coupling), which induces channel opening (Kuenze et al., 2019; Nakajo, 2019; Wu and Larsson, 2020). Unlike Shaker-like voltage-gated potassium channels (Kv1.2), the alignment of positively-charged AAs in the S4 of KCNQ1 is unique in terms of having a neutral-charge AA, glutamine (Q) at the third position (Q3) (Wu et al., 2010; Cui, 2016; Sun and MacKinnon, 2017).

We identified a novel *KCNQ1* Q234K (or Q3R) variant in the S4, a substitution for positively-charged lysine, in patients with LQTS and epileptiform activity on EEG. It was expected that an addition of a positive charge on Q3, a critical position of S4, might provide unique gating abnormalities of I_Ks_ thus resulting in unique phenotypic manifestations.

### 4.2 Interaction of KCNE1 with KCNQ1

The interaction of KCNE1 with KCNQ1 slows the channel activation rate, increases voltage-dependent current amplitude, shifts the VDA to depolarizing potentials, and eliminates channel inactivation (Barhanin et al., 1996; Sanguinetti et al., 1996; Wu and Larsson, 2020). However, how KCNE1 associates with and functionally interacts with KCNQ1 remains unknown.

KCNE1 is suggested to directly bind to the pore, particularly S6, of the KCNQ1 channels to control KCNQ1 gating, and to have contact with S4 and the extracellular end of S1 in the KCNQ1 channel (Wu and Larsson, 2020). An interaction between KCNE1 and KCNQ1 may separate the voltage-sensor movements of the I_Ks_ (WT + E1 channels) into two steps. The first step involves S4 gating charge movement, and the second step involves a slow voltage-sensor movement opening the channel. It is assumed that KCNQ1 alone channels can open after the first step, whereas WT + E1 channels only open after both steps (Barro-Soria et al., 2014; Wu and Larsson, 2020). On the other hand, Nakajo et al. assumed that there might be three states in I_Ks_ channel gating: closed state (S4 in the down state and PD closed), intermediate stage (S4 in the up state and PD closed), and open state (S4 in the full up state and PD open) (Nakajo, 2019).

When we focused on the activation rate of I_Ks_, the *KCNQ1* Q234K variant induced a marked delay in the activation rate of I_Ks_. Although the mechanism underlying the marked delay in the activation rate is unclear, there may be several possible explanations: the Q234K might affect the first step or the second step of the S4 movements, or the conformational change of the gate via VSD-PD coupling, or a combination thereof. It is also undetermined which state the Q234K affects: transition from a closed state to an intermediate state, from an intermediate state to a closed state, or a combination of the two. It is noteworthy that the electrophysiological properties of the WT + Q234K + E1 channels are generally more similar to those of the Q234K + E1 channels than the WT + E1 channels. However, the deactivation kinetics of the WT + Q234K + E1 channels resemble those of the WT + E1 channels (Figure 6), which may weaken the degree of the gain-of-function of I_Ks_. These findings suggest that the Q234K subunits in heterotetrameric channels may not equally affect the transition from the closed state to the intermediate state and the transition from the intermediate state to the open state, and *vice versa*, in the VSD-PD coupling. To clarify these matters, voltage-clamp fluorometry experiments in combination with ionic current recordings may be required.

### 4.3 Pathogenic variants and previous studies on the S4 of KCNQ1

Functional changes in the KCNQ1 channels induced by amino acid substitutions in the S4 of KCNQ1 have been reported (Table 7) (Nakajo and Kubo, 2007; Panaghie and Abbott, 2007; Shamgar et al., 2008; Wu et al., 2010). In particular, substitutions of positively-charged arginines for negatively-charged glutamic acids (R1E and R2E) in the N-terminal half (before Q3) of S4 are susceptible to becoming constitutively open in the absence and presence of KCNE1, indicating that basic residues in the N-terminal half of S4 stabilize the resting state (Wu et al., 2010). In contrast, substitutions of basic residues for glutamic acids (R4E and H5E) in the C-terminal half (after Q3) of S4 are susceptible to shifting the VDA to depolarizing potentials in the absence and presence of KCNE1, indicating that basic residues in the C-terminal half of S4 stabilize the activated state (Wu et al., 2010).

**TABLE 7:** Functional changes caused by substitutions of amino acids and disease-causing variants in S4 of KCNQ1.

Amino acid substitution in S4		without KCNE1				with KCNE1				Phenotype	References
		VDA	CD	Act rate	Deact rate	VDA	CD	Act rate	Deact rate		
R1C	R228C	D-S	Com	ND	ND	H-S	Dec	ND	ND		Nakajo and Kubo (2007)
R1Q	R228Q	H-S	ND	ND	ND	H-S	ND	ND	ND		Wu et al. (2010)
R1E	R228E	C-O	ND	ND	ND	C-O	ND	ND	ND		Wu et al. (2010)
R2W	R228W	D-S	Com	ND	ND	D-S	Dec	ND	ND		Shamgar et al. (2008)
R1A	R228A	D-S	ND	ND	ND	H-S	ND	Fast	Slow		Panaghie and Abbott (2007)
	G229D	ND	ND	ND	ND	C-O	Inc/Dec	Fast	Lost	AF	Hasegawa et al. (2014) Zhou et al. (2019)
R2C	R231C	H-S	Dec	ND	ND	Com	Dec	ND	ND		Nakajo and Kubo (2007)
R2C	R231C	ND	ND	ND	ND	C-O	Dec	ND	ND	AF, LQTS	Bartos et al. (2011)
R2Q	R231Q	C-O	ND	ND	ND	C-O	ND	ND	ND		Wu et al. (2010)
R2E	R231E	C-O	ND	ND	ND	C-O	ND	ND	ND		Wu et al. (2010)
R2K	R231K	H-S	ND	ND	ND	H-S	ND	ND	ND		Wu et al. (2010)
R2A	R231A	C-O	ND	ND	ND	C-O	Inc/Dec	ND	ND		Panaghie and Abbott (2007)
R2W	R231W	C-O	Com	ND	ND	C-O	Com	ND	ND		Shamgar et al. (2008)
Q3C	Q234C	H-S	Inc	ND	ND	H-S	Inc	ND	ND		Nakajo and Kubo (2007)
Q3R	Q234R	D-S	ND	ND	ND	D-S	ND	ND	ND		Wu et al. (2010)
Q3E	Q234E	D-S	ND	ND	ND	H-S	ND	ND	ND		Wu et al. (2010)
Q3W	Q234W	D-S	Dec	ND	ND	Com	Com	ND	ND		Shamgar et al. (2008)
Q3K	Q234K	D-S	Com	Slow	Slow/Fast	D-S	Inc/Dec	Slow	Slow	LQTS	This study
R4Q	R237Q	D-S	ND	ND	ND	H-S	ND	ND	ND		Wu et al. (2010)
R4E	R237E	D-S	ND	ND	ND	D-S	ND	ND	ND		Wu et al. (2010)
R4A	R237A	D-S	ND	ND	Slow	C-O	ND	ND	ND		Panaghie and Abbott (2007)
R4W	R237W	D-S	Dec	ND	ND	Com	Dec	ND	ND		Shamgar et al. (2008)
H5R	H240R	H-S	ND	ND	ND	D-S	ND	ND	ND		Wu et al. (2010)
H5Q	H240Q	D-S	ND	ND	ND	H-S	ND	ND	ND		Wu et al. (2010)
H5E	H240E	D-S	ND	ND	ND	D-S	ND	ND	ND		Wu et al. (2010)
	D242N	Com	Dec	Slow	ND	D-S	Dec	ND	Fast	LQTS	Moreno et al. (2017)
R6Q	R243Q	D-S	ND	ND	ND	ND	Dec	ND	ND		Wu et al. (2010)
R6E	R243E	D-S	ND	ND	ND	ND	Dec	ND	ND		Wu et al. (2010)
R6A	R243A	D-S	ND	ND	ND	Com	Dec	Fast	ND		Panaghie and Abbott (2007)
R6C	R243C	D-S	Dec	Slow	Fast	ND	Dec	ND	ND	LQTS	Franqueza et al. (1999)
R6W	R243W	D-S	Com	ND	ND	D-S	Dec	ND	ND		Shamgar et al. (2008)
R6H	R243H	Com	Com	Com	Com	D-S	Dec	Com	Fast	LQTS	Chouabe et al. (2000)

VDA, voltage dependence of activation; CD, current density; Act rate, activation rate; Deact rate, deactivation rate; D-S, depolarizing shift; H-S, hyperpolarizing shift; Com, comparable; C-O, constitutive open; Dec, decrease; Inc, increase; ND, no data; AF, atrial fibrillation; LQTS, long QT, syndrome.

Regarding neutral-charge glutamine (Q3), the effects of amino acid substitutions on the channel function appear to differ according to the substituted amino acids and the absence or presence of KCNE1 (Table 7). A substitution for cysteine (Q3C or Q234C) shifted the VDA to hyperpolarizing potentials and increased the current density in the absence and presence of KCNE1 (Nakajo and Kubo, 2007). A substitution for tryptophan (Q3W or Q234W) shifted the VDA to depolarizing potentials and decreased the current density in the absence of KCNE1, but did not affect the VDA and current density in the presence of KCNE1 (Shamgar et al., 2008). A substitution for positively-charged arginine (Q3R or Q234R) shifted the VDA to depolarizing potentials in the absence and presence of KCNE1 (Wu et al., 2010). In contrast, a substitution for negatively-charged glutamic acid (Q3E or Q234E) shifted the VDA to depolarizing potentials in the absence of KCNE1, while it shifted the VDA to hyperpolarizing potentials in the presence of KCNE1, suggesting that a positive charge at Q3 would favor the resting state (Wu et al., 2010).

Several pathogenic variants in the S4 of KCNQ1 have been reported (Table 7). *KCNQ1* G229D and R231C variants, located in the N-terminal half of S4, caused constitutively open I_Ks_ and were associated with atrial fibrillation and/or LQTS (Bartos et al., 2011; Hasegawa et al., 2014; Zhou et al., 2019). In contrast, *KCNQ1* D242N, R243C and R243H variants, located in the C-terminal half of S4, strongly shifted the VDA to depolarizing potentials or produced low I_Ks_ and are thus associated with LQTS (Franqueza et al., 1999; Chouabe et al., 2000; Park et al., 2005; Moreno et al., 2017). In the case of the R243H variant, reduced phosphaditylinositol-4,5-bisphosphate (PIP_2_) affinity of the mutant may also be involved in reduced I_Ks_ (Park et al., 2005). Like these, variants in the N-terminal half (before Q3) of S4 generally cause constitutively open I_Ks_ and are associated with atrial fibrillation and/or LQTS, whereas variants in the C-terminal half (after Q3) of S4 generally shift the VDA to depolarizing potentials thus producing low I_Ks_ and are associated with LQTS. Huang et al. reported several other variants, I227L, Q234P, L236P, and L236R in S4 associated with LQTS (Huang et al., 2018). Cell surface expression levels were not reduced, but current densities were remarkably reduced in the I227L and Q234P channels, while both the cell surface expression levels and current densities were reduced in the L236P and L236R channels.

We found that the Q3K (or Q234K) shifted the VDA to depolarizing potentials in the absence and presence of KCNE1, which resembles the effects of Q3R (or Q234R) (Wu et al., 2010), although the effects were weaker than those of the Q234R. Thus, our data support the notion that a positive charge at position 234 in KCNQ1 favors the resting state (Wu et al., 2010). Notably, we found, for the first time, that a single missense variant in the S4, Q3K (or Q234K), markedly delayed both activation and deactivation rates in the presence of KCNE1, although the activation and deactivation rates were rarely assessed in previous experiments of AA substitutions in the S4. Moreover, in the presence of KCNE1, the Q3K (or Q234K) paradoxically induced a gain-of-function during long-depolarization, even though the Q234K + E1 channels favor the resting state, whereas it induced a loss-of-function during short-depolarization (the physiological range of ventricular APD), which is due to the delayed activation rate and depolarizing shift of the VDA. Our data emphasize the importance of assessing not only the VDA but also the activation and deactivation rates of I_Ks_ to reveal the association between altered channel function and phenotypic manifestation.

### 4.4 Clinical relevance of the *KCNQ1* Q234K variant

In patients with the *KCNQ1* Q234K variant in this study, one allele contains *KCNQ1* Q234K and the other contains *KCNQ1* WT, we therefore assessed the WT + Q234K + E1 channels. The WT + Q234K + E1 channels displayed an increased current density at the end of 8-s depolarizing potentials (gain-of-function of I_Ks_), depolarizing shift of the VDA, and decelerated activation and deactivation rates. However, at 400-ms depolarizing potentials, the physiological range of the ventricular APD, the WT + Q234K + E1 channels displayed a decreased current density (loss-of-function of I_Ks_) in comparison to the WT + E1 channels. Furthermore, contrary to our expectation, the WT + Q234K + E1 channels displayed a weak frequency-dependent current accumulation, despite their delayed deactivation rates. These findings indicate that the WT + Q234K + E1 channels induce a loss-of-function of I_Ks_ during the physiological range of ventricular APD, which underlies QT prolongation. The simulation study could prove that electrophysiological properties of the WT + Q234K + E1 obtained by patch-clamp experiments can prolong ventricular APD and QT interval.

Both KCNQ1 and KCNE1 are reported to be expressed in the brain, where KCNQ1 interacts with KCNE1 (Goldman et al., 2009; Rannals et al., 2016), and there have been several reports that *KCNQ1* variants are associated with both LQTS and epilepsy (Goldman et al., 2009; Tiron et al., 2015). In addition to the LQTS phenotype, both the proband and her mother showed epileptiform activity on EEG, and her mother had been diagnosed with epilepsy, although the causes of their syncopal events are unknown. In any case, their EEGs showed apparent abnormalities compatible with epilepsy.

In the brain cells, potassium channels function as setting the resting membrane potential, reducing excitability as well as controlling the duration, shape and firing frequency of action potentials (Greene and Hoshi, 2017). Therefore, I_Ks_ loss-of-function by the *KCNQ1* Q234K variant in the brain cells may induce hyperexcitability of the brain cells. However, in case of long-lasting firings (that mimic a long-depolarization), I_Ks_ gain-of-function may act in the direction of preventing firings. It may be conceivable that the instability of membrane potential of the brain cells may be associated with epileptiform activity (or epilepsy). Moreover, the fact that *KCND3* variants which cause unique electrophysiological abnormalities (both gain- and loss-of-function) of I_to_ are associated with a novel cardiocerebral channelopathy may also suggest the association between unique electrophysiological properties of I_Ks_ and epileptiform activity (or epilepsy) (Takayama et al., 2019; Nakajima et al., 2020b).

## 5 Conclusion

We identified a novel *KCNQ1* Q234K (or Q3K) variant in the S4 of KCNQ1 in patients with LQTS and epileptiform activity on EEG. Our functional analyses revealed that the variant displayed unique gating abnormalities of I_Ks_: The variant induced an increased current density (gain-of-function) during a long-depolarization, while a decreased current density (loss-of-function) during a short-depolarization (the physiological range of ventricular APD) due to a delayed activation rate, depolarizing shift of the VDA, and weak frequency-dependent current accumulation, which can be associated with LQTS. Our data provide novel insights into the functional consequences of charge addition on Q3 in the S4 of KCNQ1.

## 6 Limitations

The association of the *KCNQ1* Q234K variant and epileptiform activity (or epilepsy) remains a matter of speculation. Further studies are needed to clarify this issue.

Regarding the simulation study, there are a few limitations. First, we failed to find the modification of any coefficients in O’Hara and Rudy model which match all experimental data in this study. In order to evaluate whether the change of parameters in the mutant channel could prolong or shorten QT interval, we focused on the important three characteristics underlying the mutant channel. Second, the V_1/2_ of activation of the Hodkin-Huxley model was 18.3 mV and was quite different as compared with 62.8 mV in the experimental data. The experimental data was conducted under the room temperature at 23°C–25°C while the human ventricle data of O’Hara and Rudy model was studied under the body temperature at 37°C. According to Q10 theory, this difference of baseline temperature could affect the kinetics of I_Ks_ current. We focused on relative differences of the channel kinetics as compared with WT because the purpose of the simulation study in this study was to elucidate whether the Q234K model corresponding to the experimental study could prolong or shorten the APD or QT interval as compared with WT.

## Data Availability

The datasets for this article are not publicly available due to concerns regarding participant/patient anonymity. Requests to access the datasets should be directed to the corresponding author. The original contributions presented in the study are publicly available. These data can be found here: https://gnomad.broadinstitute.org/gene/ENSG00000053918?dataset=gnomad_r4, https://www.ncbi.nlm.nih.gov/clinvar/?term=KCNQ1%5Bgene%5D&redir=gene, https://sift.bii.a-star.edu.sg/, http://genetics.bwh.harvard.edu/ggi/pph2/a6b96a0ed66a5f1c4a4af7e58c3eebd9138f4989/9873417.html.
